# Feasibility of Implant Strain Measurement for Assessing Mandible Bone Regeneration

**DOI:** 10.3390/mi13101602

**Published:** 2022-09-27

**Authors:** René Marcel Rothweiler, Sergej Zankovic, Leonard Simon Brandenburg, Marc-Anton Fuessinger, Christian Gross, Pit Jacob Voss, Marc-Christian Metzger

**Affiliations:** 1 Department of Oral and Maxillofacial Surgery, Faculty of Medicine, University of Freiburg, 79106 Freiburg, Germany; 2 G.E.R.N. Center for Tissue Replacement, Regeneration & Neogenesis, Faculty of Medicine, Albert-Ludwigs-University of Freiburg, 79108 Freiburg, Germany

**Keywords:** bone regeneration, mandible reconstruction, fracture, strain measurement, nonunion

## Abstract

Nonunion is one of the most dreaded complications after operative treatment of mandible fractures or after mandible reconstruction using vascularized and non-vascularized bone grafts. Often diagnosis is made at advanced stage of disease when pain or complications occur. Devices that monitor fracture healing and bone regeneration continuously are therefore urgently needed in the craniomaxillofacial area. One promising approach is the strain measurement of plates. An advanced prototype of an implantable strain measurement device was tested after fixation to a locking mandible reconstruction plate in multiple compression experiments to investigate the potential functionality of strain measurement in the mandibular region. Compression experiments show that strain measurement devices work well under experimental conditions in the mandibular angle and detect plate deformation in a reliable way. For monitoring in the mandibular body, the device used in its current configuration was not suitable. Implant strain measurement of reconstruction plates is a promising methodical approach for permanent monitoring of bone regeneration and fracture healing in the mandible. The method helps to avoid or detect complications at an early point in time after operative treatment.

## 1. Introduction

**Figure 1:**
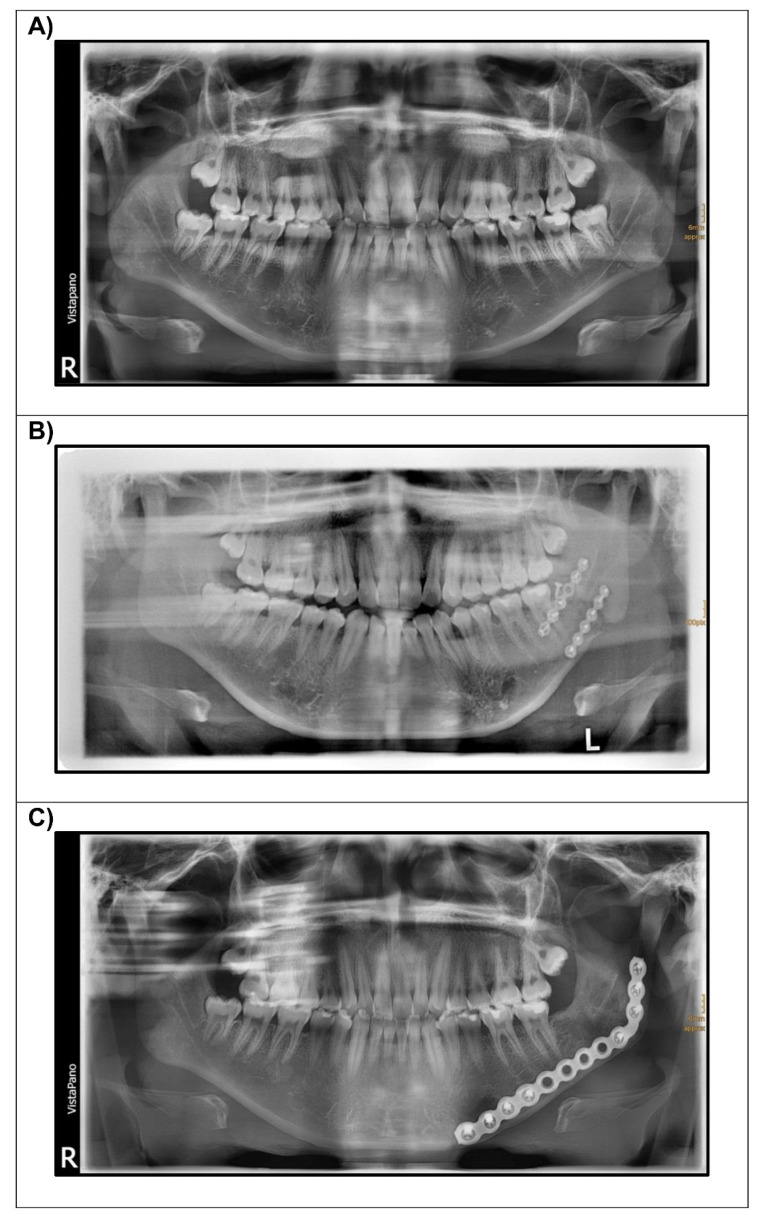
Exemplary patient case of secondary reconstruction using a 2.8 mm reconstruction plate after failure of primary fracture fixation with two miniplates (panoramic radiographs (**A**) after trauma; (**B**) after reconstruction with two miniplates; (**C**) after secondary reconstruction using a reconstruction plate).

Nonunion is one of the most dreaded complications that might arise after mandible fractures. It is defined as absence of osseous bridging of the fracture eight weeks after treatment [1,2]. The incidence of this complication is <5%. It can also occur after mandible reconstruction using vascularized and non-vascularized bone grafts in ablative surgery, however, here the incidence is slightly higher [3]. Most of the cases are caused by postoperative trauma or osteomyelitis. Treatment delay in polytrauma patients or due to other reasons does not lead to a higher incidence of nonunion [4,5]. Furthermore, removal of impacted third molars in fracture lines in the mandibular angle does not result in a significant difference with regard to the occurrence of nonunions [6]. To avoid nonunion, a stable fixation of the fracture is important, preferably with two miniplates [7]. A rigid fixation with one single reconstruction plate applied via an extraoral approach is an alternative that can be used in patients being noncompliant with instructions, oral hygiene and follow-up [8]. It can also be used for secondary reconstruction if a first attempt of fracture fixation with two miniplates was not successful (Figure 1).

Standard examination method for monitoring fracture and bone healing after surgical procedures is radiography in addition to clinical examination. Here, panoramic radiographs and CBCT radiographs are the favored methods. However, radiation exposure which ranges from 10 µSv (panoramic radiograph) to 1200 µSv (CBCT) should not be neglected and imaging should only be carried out when indispensable (annual background radiation 2.000–4.500 µSv) [9]. Routine continuous monitoring is therefore not possible. Other radiation-free imaging modalities, such as ultrasound, have been described in experimental settings but are still experimental and have not yet found their way into the clinical setting [10].

New methods for monitoring fracture healing and bone regeneration are therefore urgently needed. One promising approach, which has been developed since the 1970s in long bones, is strain measurement on metallic implants [11]. The sensors which are commonly mounted on or integrated in nails or locking plates measure deformation when forces are applied to implants. Prerequisite is a load-bearing osteosynthesis. The more the fracture heals or the bone regenerates, the less force is applied on the plate or the nail which results in a lower implant deformation and a lower deflection of the signal that can be achieved. To date, numerous active and passive devices have been developed and described but none of them have found their way into clinical use [12].

**Figure 2:**
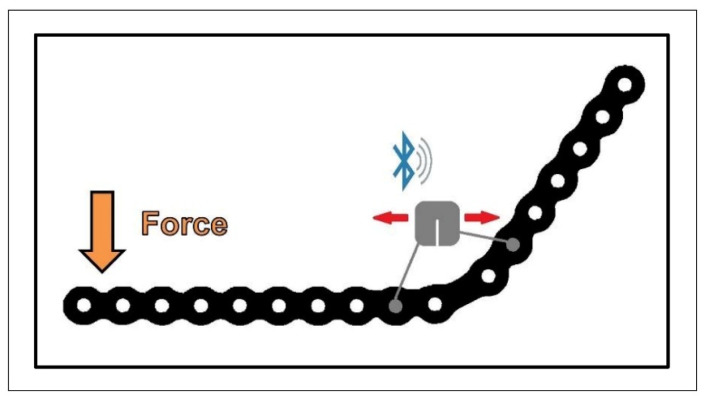
Schematic image of the functionality of the strain sensor.

The following experiments were intended to investigate the feasibility of this method for a possible future indication in the maxillofacial area, especially in the mandible. We chose an advanced prototype of strain sensor designed for use in long bones and spine surgery that had already been successfully tested in various animal models [13,14]. The device records bending and stress on the housing, and thus, indirectly on the plate surface on which it is mounted. Collected data is transmitted via a Bluetooth connection; the device is an “active” device and contains a battery inside the housing for power supply [12,15,16] (Figure 2).

## 2. Materials and Methods

### 2.1. Strain Sensor

The housing of the sensor is made of titanium grade 5, openings are hermetically sealed by laser melting. The sensor is L-shaped with the larger electronic parts placed in a bulge next to the plate on which the device is mounted. The thickness of the sensor device on the plate is therefore less than 2.5 mm. A strain-gauge Wheatstone half-bridge is incorporated for strain measurement, the sampling rate is 10 Hz after amplification of the signal. Internal data processing is performed by a power-saving microprocessor. An additional integrated low-power accelerometer is used to control wake-up and sleep times of the microprocessor according to the movement of the plate on which the sensor is mounted. For power supply, a Li-Ion battery is installed in the housing [14] (Table 1).

### 2.2. Data Processing

Initial data storage was performed by transferring data via Bluetooth to the investigator’s smartphone. After transmission to the existing cloud, the final analysis was carried out using a Microsoft Excel spreadsheet (Microsoft Excel^®^ Version 16.0, Microsoft Corporation, Albuquerque, NM, USA). Representative graphs were generated over a period of 180 s.

### 2.3. Experimental Setups

**Figure 3:**
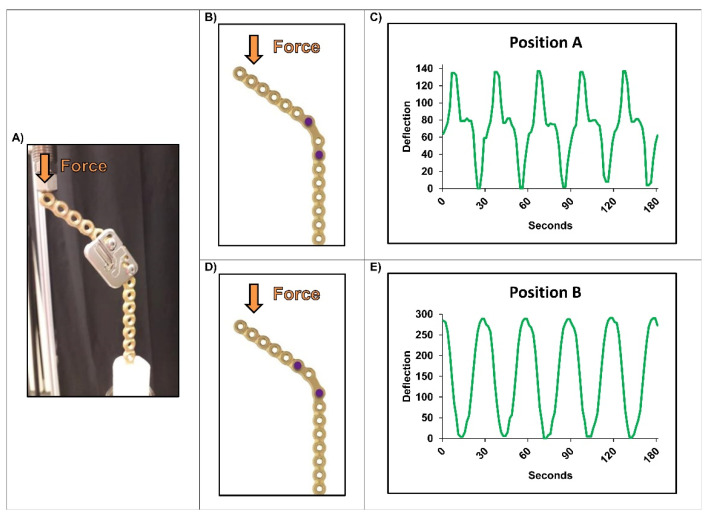
First compression experiment of the strain measurement device mounted to a 2.8 mm Matrix Mandible plate (**A**): (**B**,**C**) Insufficient detection of a sinusoidal movement (displacement 1.5 mm; f = 0.5 Hz), when mounting device in Position A; (**D**,**E**) Perfect registration of the sinusoidal movement (displacement 1.5 mm; f = 0.5 Hz; no defined force), when mounting device in Position B.

Two different experiments were performed. In the first setup a non-bended Matrix Mandible 2.8 mm reconstruction plate (DePuySynthes; REF No. 04.503.772) was embedded in PMMA on the longer side. The embedded plate was fixed with the longer side perpendicularly to a MTS Mini Bionix Compression machine. The strain sensor was mounted with two M3 screws and nuts to the plate in two different ways [Position A/B] (Figure 3). A compression pattern was then applied to the plate with a sinusoidal command of 1.5 mm displacement at 0.5 Hz and undefined force.

In the second more physiologic setup the same Matrix Mandible 2.8 mm reconstruction plate was bent to a synthetic mandible (Synbone; REF No. 8900) as performed before [17,18]. This type of mandible was selected as the different specimens are identical in dimensions and other characteristics and match the human mandible. After creating an artificial fracture in the form of a segmental defect in two different regions (two different bones), the defect was bridged using the pre-bent plate with four 2.9 mm locking screws (DePuySynthes; 14 mm length; REF No. 04.503.674.04C) bicortically on each side. The strain sensor was mounted again with two M3 screws and nuts to the mounted plate. In the mandibular angle, the sensor could only be placed in the unfavorable “Position A” due to the lack of space and the required fixation of the plate with four screws in the ascending ramus. The fixated mandibles were placed on a Zwick/Roell compression machine (Zwick/Roell Amsler HC-10). A similar compression pattern was then applied to the mandible as before, exerting a sinusoidal force to the incisors (1.5 mm displacement at 0.5 Hz; no defined force).

## 3. Results

In the first experiment the strain sensor was mounted to the reconstruction plate in the mandibular angle in two different positions. The sinusoidal compression on the plate with a frequency of 0.5 Hz caused a visible deformation of the plate; a registration of this pressure could be obtained with the monitor in both regions. “Position B” (Figure 3D,E) as mounting space proved as superior compared to “Position A” (Figure 3B,C). The curve recorded here had a higher deflection overall and was more sinusoidal compared to the one in “Position A” where a plateau was visible.

**Figure 4:**
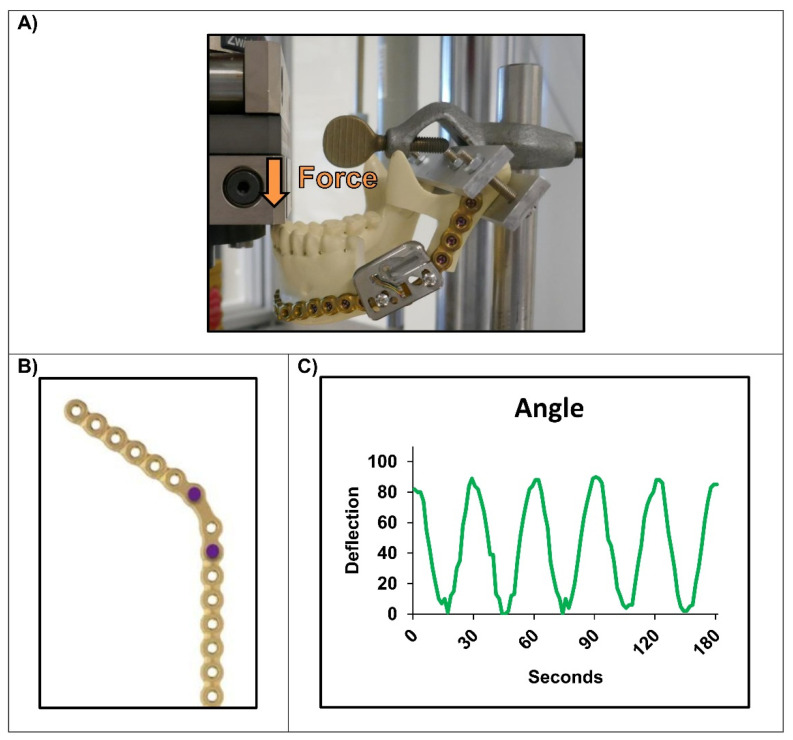
Simulation of the mounted strain measurement device on an artificial defect in the mandibular angle (displacement 1.5 mm; f = 0.5 Hz): (**A**) Mounted sensor to the mandible; (**B**) Used holes for fixation of the device; (**C**) Nearly perfect registration of the sinusoidal force being applied to the incisors.

In the more physiological experiments the strain sensor was first attached to a bridged defect in the mandibular angle of the synthetic mandible. Compression experiments with approximately the same parameters as before (1.5 mm displacement at 0.5 Hz, no defined force) and force application to the incisors of the mandible also showed a good detection of the applied force although the monitor could only be placed in the unfavorable “Position A” (Figure 4).

**Figure 5:**
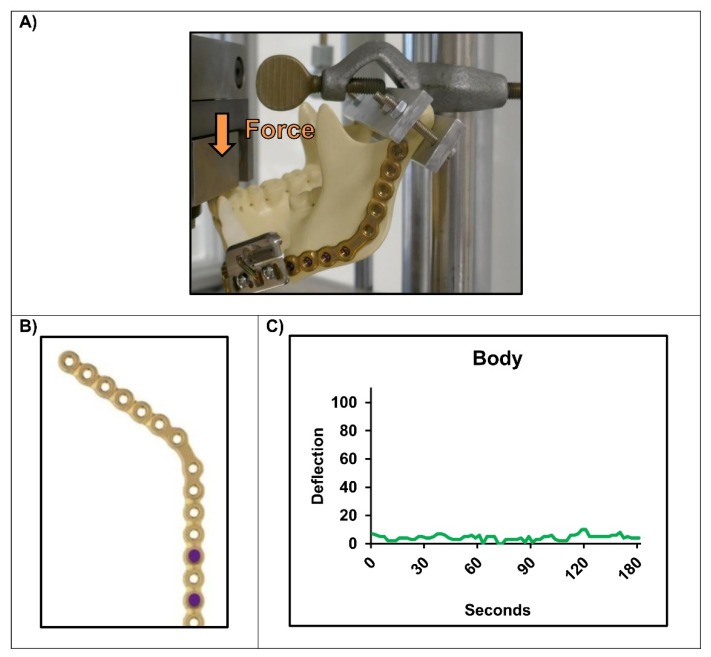
Simulation of the mounted strain measurement device on an artificial defect in the mandibular body (displacement 1.5 mm; f = 0.5 Hz; no defined force): (**A**) Mounted sensor to the mandible; (**B**) Used holes for fixation of the device; (**C**) No registration of the sinusoidal force being applied to the incisors.

After attaching the strain sensor to the mandibular body, no deflection of the recorded curve was detected at all with the same parameters as in the previous experiment (1.5 mm displacement at 0.5 Hz, force applied to the incisors) (Figure 5). Even increasing the displacement up to fracture of the condylar heads did not improve this result (data not shown). 

## 4. Discussion

Smart implants for monitoring of bone regeneration and fracture healing have been developed since the 1970s [11]. They are usually based on the functional principal of recording implant deformation in long bones (strain measurement) and are mounted to load-bearing implants such as plates or nails. The more advanced the bone regeneration the less force is transmitted through the implant, resulting in less deformation of the implant and thus less displacement of a plotted curve [15,19]. Our aim was to investigate implant strain measurement for its potential applicability in the maxillofacial region since in today’s clinical environment only clinical examination and radiographic procedures are available as diagnostic methods.

Mounting the strain sensor in the mandibular angle on a 2.8 mm reconstruction plate resulted in a deflection of the recorded curve in both of our setups. However, the quality of this curve was different depending on the fixation points. In contrast, a registration of the deflection was not possible in the mandibular body. This finding can be easily explained by finite element analysis and other studies that demonstrated high tensile loading and deformation of mandibular reconstruction plates in the mandibular angle when compared to the mandibular body [18,20,21]. The lower deformation of the mandibular body results in insufficient registration by the sensor when positioned in this area. Since the used prototype of the strain sensor has been designed only for the detection of forces along the longitudinal axes due to its intended purpose (it measures bending and tension on the plate surface), it is also not capable in its current form of differentiating the multidimensional distribution of forces that occur in the mandible [22,23]. Modifications of future strain sensors, possibly with integration of a multidimensional force registration or also adaptation of the resolution, could further increase the informative value in the context of fracture healing or also new bone formation for later use in the maxillofacial region, particularly in the mandibular body.

Today’s strain sensor products and prototypes are certainly too large for an immediate implementation in the field of craniomaxillofacial surgery. The main reason for this circumstance are the batteries that have to be installed inside the housing of strain measurement sensors to provide the necessary energy for the wireless connection needed for permanent data transmission (“active device”). By changing the transmission protocol to NFC or RFID, it is possible to develop sensors without a battery (“passive devices”) however, the inductive coils available today are quite large compared to a mandible reconstruction plate and the penetration depth of the signal into soft tissue is quite low [24]. Furthermore, recording can only be achieved when the receiver is placed next to the coil; a continuous measurement is hardly possible anymore [25,26]. Nevertheless, these techniques seem to be a promising option for devices in the maxillofacial region as induction coils are constantly being developed and, if desired, they can also be incorporated directly into the plate with the strain sensor.

Other completely different technical methods such as ultrasound or the measurement of bite forces at different time points are also still experimental approaches for fracture monitoring and have not been implemented in a clinical setting either [10,27,28,29]. Unlike strain sensors, the results and quality of the data obtained depend on the investigators and their experience in using these methods; moreover, they do not allow continuous monitoring like the “passive devices” or X-ray examinations mentioned above.

Taken together, implant strain measurement represents a promising approach for measuring bone regeneration and fracture healing in the maxillofacial area. “Active” strain measuring devices with built-in battery allow continuous monitoring without interruptions, while intermittent monitoring is possible with “passive” protocols such as NFC or RFID. Complications may be diagnosed earlier, leading to a better and safer outcome in complicated mandible fractures or complex reconstructions with grafts. In addition to that, implant strain measurement could help us to gain further information about the physiology and efficiency of bone healing in experimental settings such as testing new scaffolds in animal models [30,31,32]. However, future devices will require many adjustments and optimizations (e.g., downsizing; multidimensional force registration) before they can be used in craniomaxillofacial surgery.

## 5. Conclusions

Implant strain measurement of reconstruction plates seems to be an appropriate method for registration and monitoring of bone regeneration and fracture healing in the mandible. By applying forces to a load-bearing osteosynthesis plate, deformation in the plate and thus the indirect healing of the bone is measured. Depending on the transmission protocol used, data can be transmitted continuously (“active devices” with Bluetooth connection) or interrupted (“passive devices” with NFC or RFID connection). If developed further in the future, these devices could help prevent or detect complications early after surgical fracture treatment or reconstructive surgery. Furthermore, with their help we could gain new insights into the physiology of bone healing in the maxillofacial region.

## Figures and Tables

**Table 1:** The characteristics of the strain-gauge device.

Type of Sensor	Active; Strain-Gauge Wheatstone Half-Bridge
Battery	Li-Ion
Housing	Titanium grade 5
Sampling rate	10 Hz
Transmission protocol	Bluetooth

## Data Availability

All data can be requested from the corresponding author.
